# Predicting suitable habitats of *Melia azedarach* L. in China using data mining

**DOI:** 10.1038/s41598-022-16571-y

**Published:** 2022-07-23

**Authors:** Lei Feng, Xiangni Tian, Yousry A. El-Kassaby, Jian Qiu, Ze Feng, Jiejie Sun, Guibin Wang, Tongli Wang

**Affiliations:** 1 Co-Innovation Centre for Sustainable Forestry in Southern China, College of Forestry, Nanjing Forestry University, Nanjing, 210037 China; 2 Department of Forest and Conservation Sciences, Faculty of Forestry, University of British Columbia, Vancouver, BC V6T 1Z4 Canada; 3 School of Mathematics and Statistics, Yunnan University, Kunming, 650504 China; 4 College of Pharmacy, Kangda College of Nanjing Medical University, Lianyungang, 222000 China; 5 College of Biology and the Environment, Nanjing Forestry University, Nanjing, 210037 China

**Keywords:** Agroecology, Climate-change ecology, Ecological modelling, Forestry, Climate change, Agroecology, Climate-change ecology, Ecological modelling, Forestry

## Abstract

*Melia azedarach* L. is an important economic tree widely distributed in tropical and subtropical regions of China and some other countries. However, it is unclear how the species’ suitable habitat will respond to future climate changes. We aimed to select the most accurate one among seven data mining models to predict the current and future suitable habitats for *M. azedarach* in China. These models include: maximum entropy (MaxEnt), support vector machine (SVM), generalized linear model (GLM), random forest (RF), naive bayesian model (NBM), extreme gradient boosting (XGBoost), and gradient boosting machine (GBM). A total of 906 *M. azedarach* locations were identified, and sixteen climate predictors were used for model building. The models’ validity was assessed using three measures (Area Under the Curves (AUC), kappa, and overall accuracy (OA)). We found that the RF provided the most outstanding performance in prediction power and generalization capacity. The top climate factors affecting the species’ suitable habitats were mean coldest month temperature (MCMT), followed by the number of frost-free days (NFFD), degree-days above 18 °C (DD > 18), temperature difference between MWMT and MCMT, or continentality (TD), mean annual precipitation (MAP), and degree-days below 18 °C (DD < 18). We projected that future suitable habitat of this species would increase under both the RCP4.5 and RCP8.5 scenarios for the 2011–2040 (2020s), 2041–2070 (2050s), and 2071–2100 (2080s). Our findings are expected to assist in better understanding the impact of climate change on the species and provide scientific basis for its planting and conservation.

## Introduction

*Melia azedarach* L. (Meliaceae) is a fast-growing species with good timber attributes of multiple-use, such as construction and furniture, farm tools, boats, vehicles, and musical instruments manufacturing^1^. The species roots, bark, flowers, and fruits are of high medicinal values^2,3^. Additionally, its fruit and leaf extracts can control numerous agricultural pests and are commonly used as biological pesticides raw materials^4^. The species is an excellent urban greening tree that is resistant to smoke and dust and can absorb many toxic and harmful gases. In China, it is widely distributed in mixed forests, fields, and roadsides between 18 and 40° N of China, occupying about one-third of the country's land area^5^. It it is mainly used in plant pesticides, timber, medicinal and ecological restoration^6^. However, uncertain climate factors may reshape their future suitable habitats in China^7^.

The intensification of global warming, accompanied by the frequent occurrence of extreme natural disturbances, such as wind storms, droughts, fires, and floods, will undoubtedly impact the global forest ecosystem^8^. Different tree species respond differently to climate change, with positive and negative effects in different areas. For example, climate change is expected to increase the suitable habitats of Mediterranean oaks in the western temperate areas^9^ as well as the total suitable habitat for *Cypripedium japonicum*^10^. Conversely, *Eucalyptus* species are expected to face future challenges due to their poor spread capability^11^, and Persian oak (*Quercus macranthera*) will experience a reduction in its contemporary range and is expected to move to higher altitudes^12^. Consequently, assessing the impact of climate change on the potential suitable habitat of species and formulating sustainable forest management strategies are critical to maintaining forest ecosystems integrity.

With climate change challenges, species distribution models (SDMs) have become essential tools for projecting plants adaptation to a changing climate^11^. At present, a variety of data mining techniques have been applied to model species distribution data. For example, GLM was used to predict the spread of Emerald Ash Borer (*Agrilus planipennis*) in southern Ontario, Canada^13^. The NBM predicts the potential distribution areas of *Taxus chinensis* and identifying plant long non-coding RNA and predicting its functions^14^. Hailu Shiferaw et al. selected the best performing algorithm for mapping the coverage of *Prosopis juliflora* (Swartz DC.) in Afar, Ethiopia by comparing GBM, RF and SVM algorithms^15^. Maxent models have been widely used in the fields of crop niches, plant diseases and insect pests, and species invasion prediction^16,17^. Therefore, we compared these modeling approaches through data mining techniques to identify the most effective modeling approach to predict the suitable habitat of *M. azedarach* species distribution based on the relationship between its occurrence and climate variables.

Understanding the potential impact of climate change on the suitable habitat of *M. azedarach* is of great significance to its cultivation and conservation in China. Studies conducted on *M. azedarach* were mainly focused on tree and stand productivity, extraction of active ingredients, and pest resistance potential^3,18^. Research on *M. azedarach* potential distribution as affected by climate change is lacking and thus, the present study is aimed at exploring the above-mentioned seven data mining techniques to establish climate-based distribution prediction models and select the best model in predictions of the species future suitable habitat. Our specific objectives were to: (1) compare the prediction accuracy of the seven modeling algorithms and select the one with the best performance; (2) determine the key climatic factors related to suitable habitat; (3) develop current and future suitable habitat maps for *M. azedarach* in China and highlighting the areas of change; and (4) assess the potential impact of future climate change on the species suitable habitat.

## Material and methods

### Species location data

**Figure 1:**
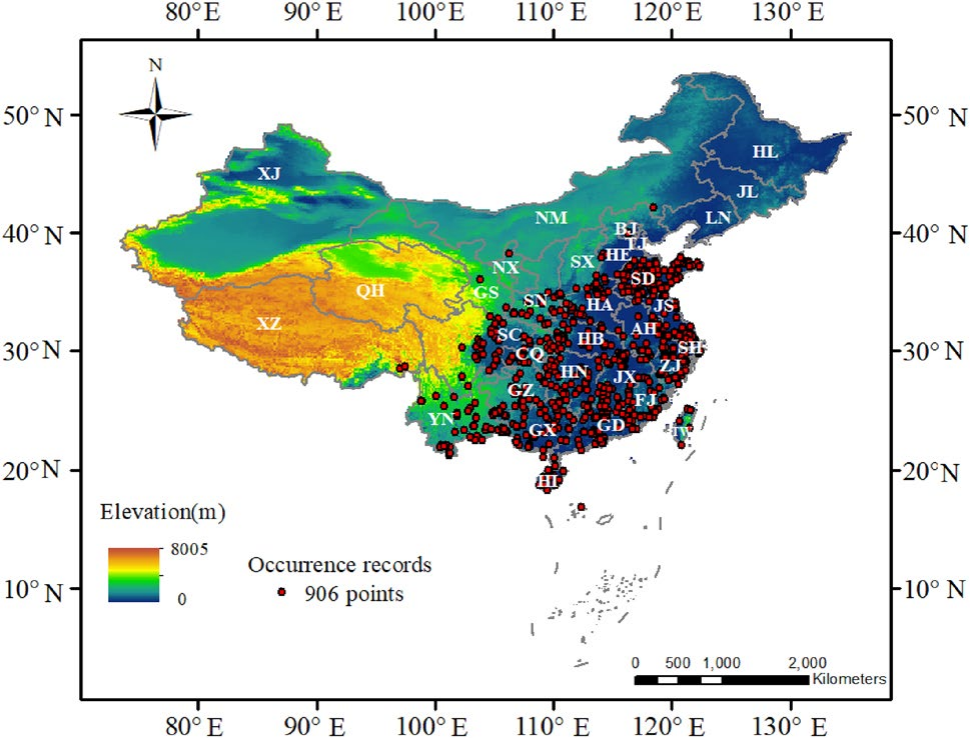
Distributions of the 906 *M*. azedarach occurrence records. The map was created in ArcMap 10.2 of the Environmental System Resource Institute, Icn. (https://www.esri.com/zh-cn/arcgis/products/arcgis-desktop/resources).

Here, we used the Chinese presence and absence *M. azedarach* data to establish the prediction models. First, we found 1,432 presence data (data source: Global Biodiversity Information Facility (GBIF), https://doi.org/10.15468/dl.3t8r62, accessed on 17 May 2022, and the Chinese Virtual Herbarium (CVH), http://www.cvh.ac.cn/, accessed on 17 May 2022). All the *M. azedarach* distribution data have been licensed. To avoid redundant sampling, we deleted those sample points with similar longitude and latitude^19^. Then a 0.01° mesh thinning was performed, and the actual distance corresponding to 0.01° was about 1 km and only one distribution point was reserved in each grid so that the distance between sample points was more than 1 km^20^. A total of 906 samples were included for model building. Finally, we used ArcMap 10.2 to overlay the asc result file generated by the model with the map of China to generate the final result map (Fig. 1). In addition, all maps in our study were created in ArcMap 10.2.

### Environment variables

We used *M. azedarach* presence-absence data as the dependent variable and 16 climatic factors derived from ClimateAP_v221 software (http://ClimateAP.net) as predictors to build the model (Table S1)^21^. We used the following tests to avoid the effect of multicollinearity among the climate variables. First, the variance inflation factor (VIF) was calculated for each of the 16 variables (Table S2). Second, the correlation analysis was conducted for each pair of the 16 variable (Figure S1). Finally, we used stepwise regression analysis to eliminate the variables that led to an observed multicollinearity^22^.

### Model development and prediction

**Figure 2:**
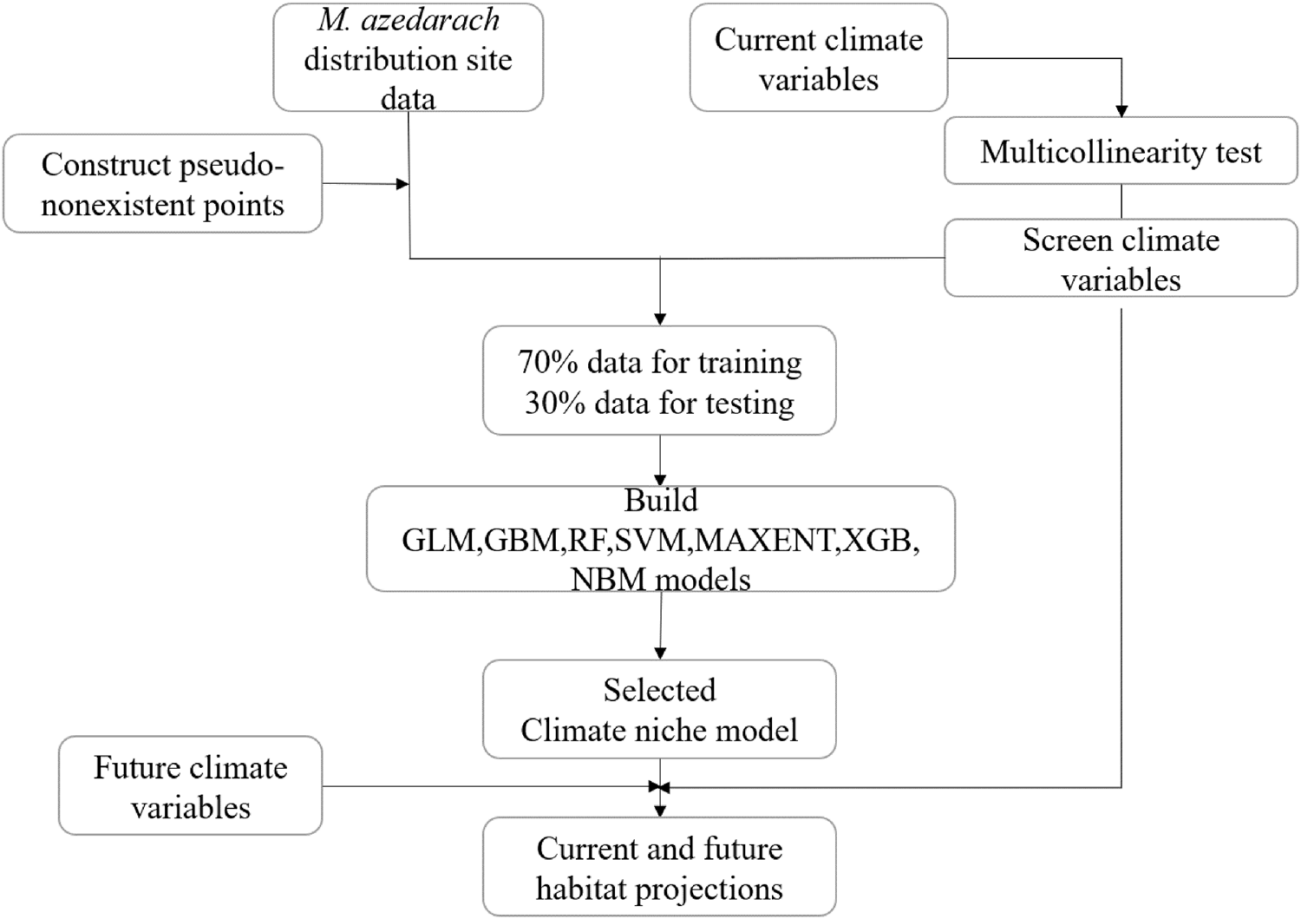
Flowchart for modeling of *M. azedarach*.

We used seven models (Generalize Linear Model (GLM), Gradient Boosting Machine (GBM), Random Forest (RF), Support Vector Machine (SVM), Maximum Entropy (MaxEnt), Extreme Gradient Boosting (XGBoost), and Naive Bayesian Model (NBM)) to associate the distribution of *M. azedarach* with climate variables. We used a data-driven approach to select the number of pseudo-existent points, and started with 1000, 2000, 10,000, 30,000, and 100,000 pseudo-nonexistent points. It was found that the most models had the highest prediction accuracy with 2000 pseudo-nonexistent points. Therefore, we used the “dismo” package in R to randomly generate 2000 “pseudo-nonexistent” records in the study area. Models were established with species presence-absence data as the dependent variable and climate variable as the independent variables. In order to evaluate the models’ prediction accuracy, we randomly selected 70% data for training and the remaining 30% data for testing (validation). We used the “caret” package to train and adjust the parameters for all the seven models except Maxent, since it facilitates the process of building, evaluating, as well as selecting features. Then, ten cross-verifications were carried out, and each model was repeated 10 times. At the same time, the Maxent model was executed using the Maxent version 3.4.4 software in R-package (Fig. 2).

### Model validation

To assess the performance of the seven predictive models, we compared their area under receiver operating character curve (AUC), Kappa, and overall accuracy (OA). The AUC is the probability value, with evaluation criteria were: 0.5–0.6 = fails, 0.6–0.7 = poor, 0.7–0.8 = fair, 0.8–0.9 = good, 0.9–1.0 = excellent^23^. Kappa coefficient is an index to measure classification accuracy. The calculation result of kappa is − 1 to 1, but usually, kappa falls between 0 and 1, which can be divided into five groups: 0.0–0.2 means very low consistency, 0.21–0.40 means general consistency, 0.41–0.60 means moderate consistency, 0.61–0.80 means high consistency, 0.81–1 means almost perfect^24^. Both Kappa and AUC consider the true positive rate and true negative rate to avoid an overestimation or underestimation error (Sahin 2020).

### Habitat classification

Appropriate habitat evaluation index values were determined as follows: predicted values of 0–0.2, 0.2–0.4, 0.4–0.6, and > 0.06 were deemed unsuitable, low-, medium-, and high-suitable habitat, respectively^25^ (All methods were performed in accordance with the relevant guidelines and regulations).

## Results

### Models performance evaluation

**Figure 3:**
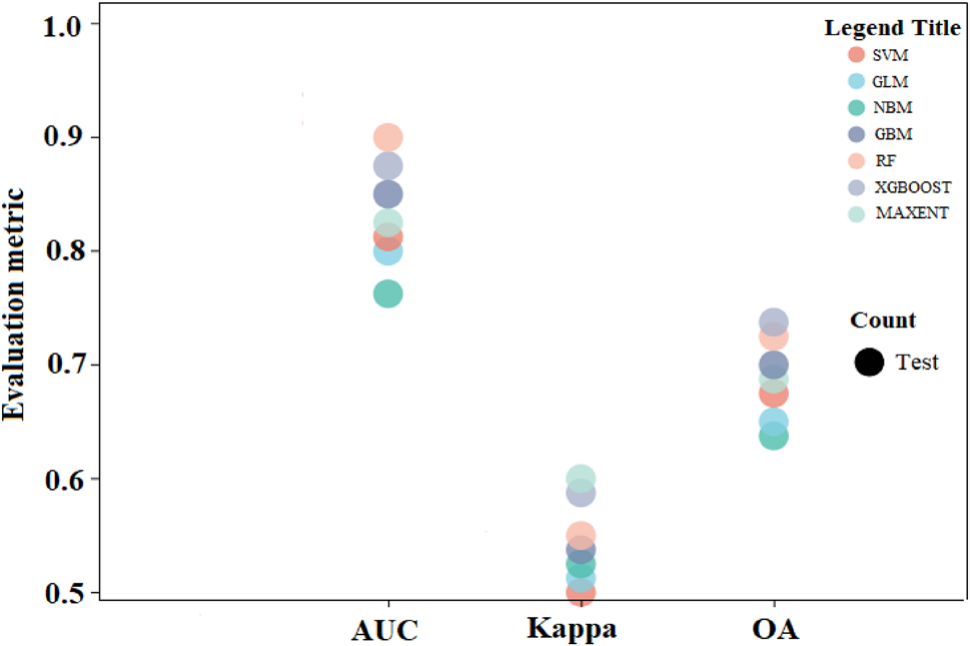
Bubble diagram of evaluation metrics for testing data. Different color bubbles represent different models.

Through the cross-validation evaluation of the tested models, Kappa, AUC, and OA values were obtained for the testing portions of the dataset (Fig. 3). All models performed well (AUC > 0.8, Kappa > 0.5, and OA > 0.7). The AUC values of the seven models varied from 0.85 (NBM) to 0.90 (RF), Kappa values varied from 0.53 (SVM) to 0.59 (MaxEnt), and Overall accuracy values ranged from 0.77 (NBM) to 0.81 (XGBoost). Overall, the three evaluation metrices all indicated that the Random Forest (RF) model provided the best predictive performance and while the Naive Bayesian Model (NBM) was the worst, thus, we selected the RF model to establish *M. azedarach* distribution patterns.

### Important climate variables and their response curves in random forest (RF)

Through multicollinearity analysis of the variables, we finally identified ten key climate variables. The top three climate variables contributing to the RF model include MCMT (189.24), NFFD (180.69), and DD > 18 (104.77), followed by TD (72.82), MAP (69.43), DD < 18 (64.12), DD > 5 (56.27), and AHM (54.88); and finally DD < 0 (44.01) and PAS (28.54) also played some roles in the determining the potential distribution of *M. azedarach* (Table 1).

**Table 1:** Contributions of the most influencing climate variables to the *M. azedarach *random forest (RF) model.

Variable^1^	Units	Overall contribution
MCMT	°C	189.24
NFFD	day	180.69
DD > 18	°C-days	104.77
TD	°C	72.82
MAP	mm	69.43
DD < 18	°C-days	64.12
DD > 5	°C-days	56.27
AHM		54.88
DD < 0	°C-days	44.01
PAS	mm	28.54

^1^See Table S1 for variables abbreviations.

**Figure 4:**
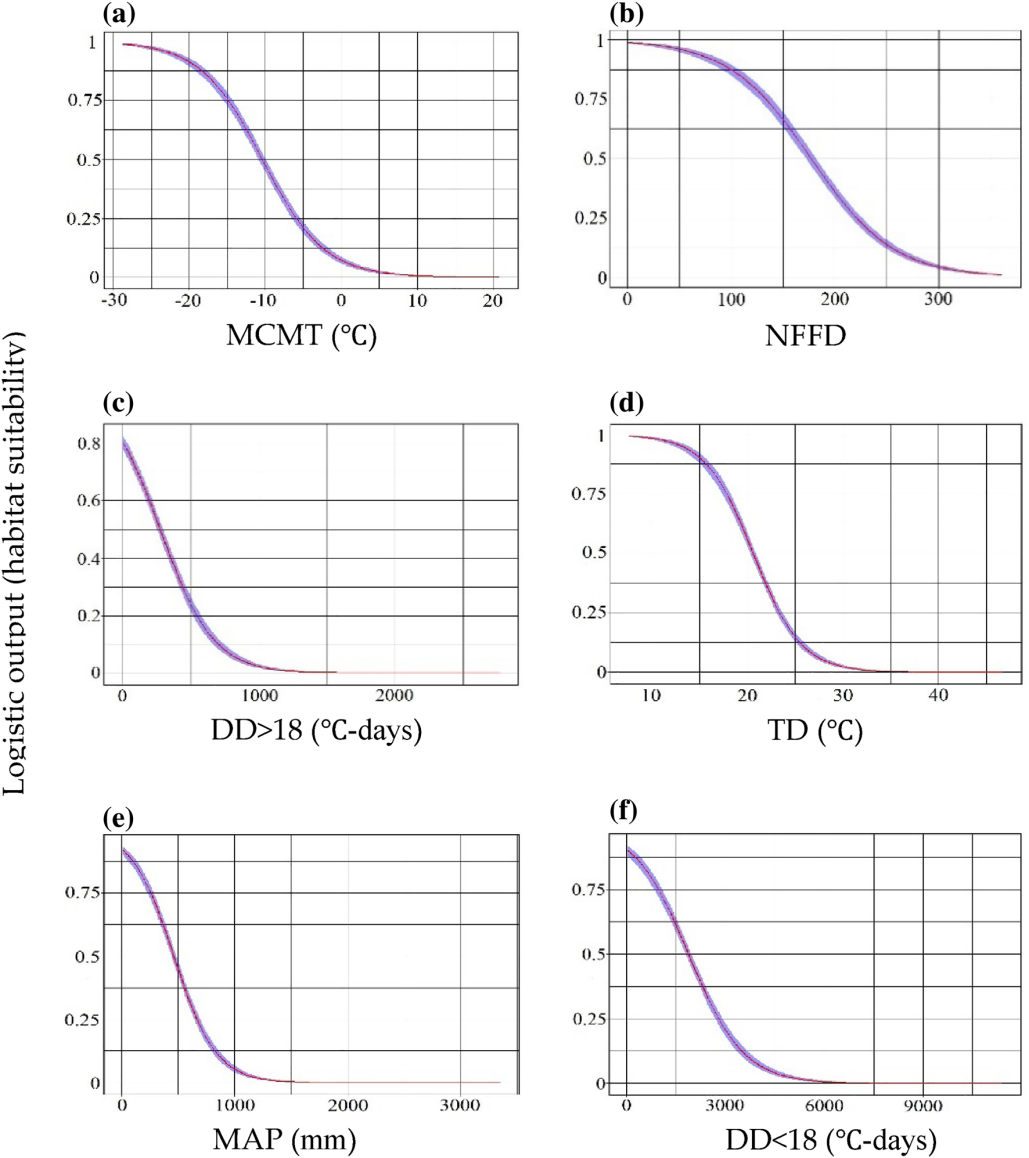
Response curves of the top six important climate variables (**a**–**f**) in the RF model. When the logical output > 0.5, the probability of species presence under this condition is higher than that under a typical condition, indicating that the condition is suitable for tree species.

Figure 4 displayed the relationships between the top six climate variables and *M. azedarach* suitability according to the predictions of RF algorithms. The habitat suitable range was between − 10 and − 28 °C for MCMT (Fig. 4a), between 0 and 175 days for NFFD (Fig. 4b), between 0 and 250 for DD > 18 (Fig. 4c), between 5 and 21℃ for TD (Fig. 4d), between 0 and 480 mm for MAP (Fig. 4e), and between 0 and 1750 for DD < 18 (Fig. 4f).

### RF model prediction of *M. azedarach* contemporary habitats distribution

**Figure 5:**
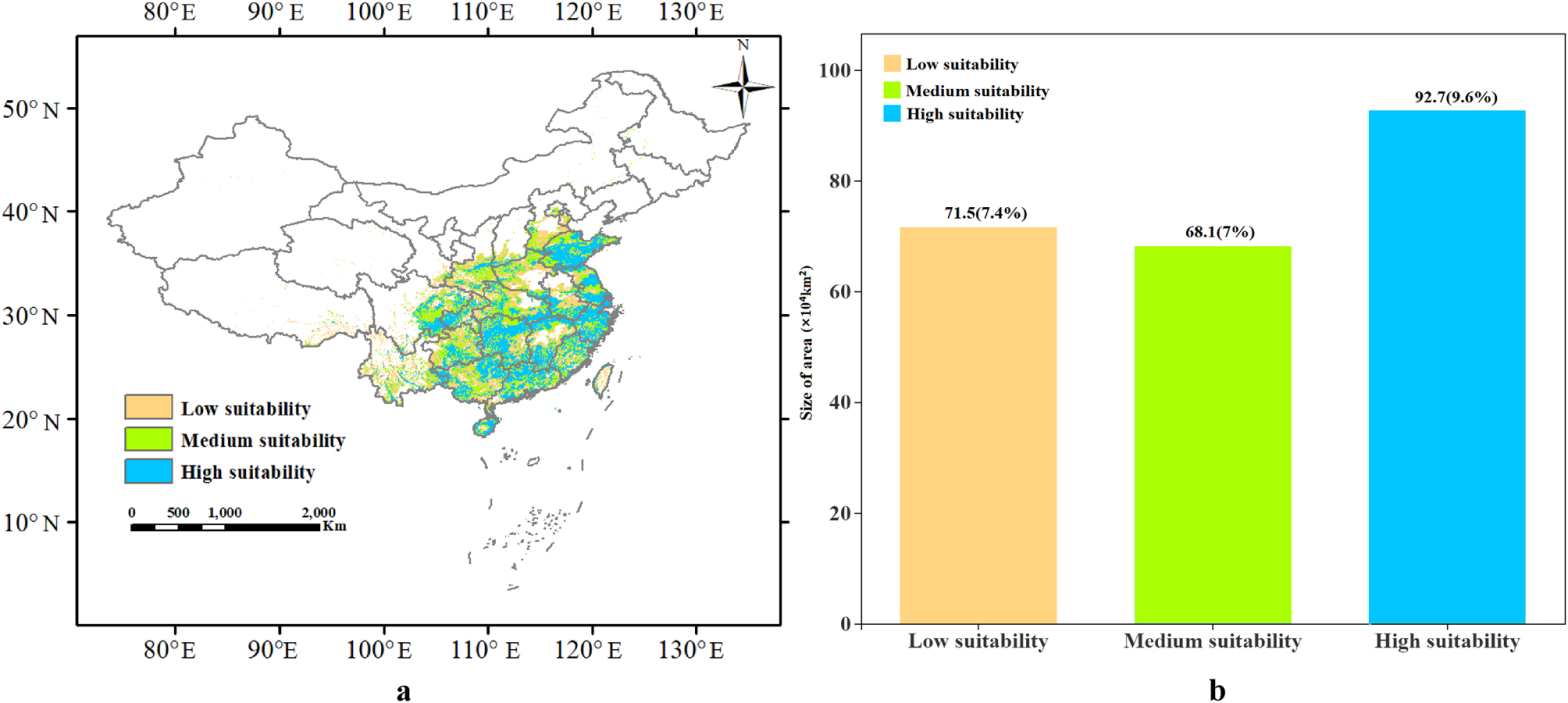
(**a**) *M. azedarach* contemporary suitable habitats distributions (1960–1990) and (**b**) their percentage representations. The map was created in ArcMap 10.2 of the Environmental System Resource Institute, Icn. (https://www.esri.com/zh-cn/arcgis/products/arcgis-desktop/resources).

The spatial distributions of *M. azedarach* and areas of suitable habitats under current climatic conditions as predicted by the RF algorithm are shown Fig. 5. The overall suitable habitat was mainly distributed between 18 and 40° N (Fig. 5a). These habitats were classified as: (1) high-suitable habitats (mainly scattered in Shandon (SD), Jiangsu (JS), Shanghai (SH), Zhejiang (ZJ), Guangdong (GD), Hunan (HN), Hainan (HI), South Jiangxi (JX), the junction of the three provinces of Hubei (HB), Anhui (AH), Jiangxi (JX), and the junction of Chongqing (CQ) and Sichuan (SC), covering 9.3 × 10^5^ km^2^ (9.6%; Fig. 5b); (2) medium-suitable habitats (scattered around the high-suitable habitats, covering 6.8 × 10^5^ km^2^ (7%; Fig. 5b) and specifically concentrated in eastern Sichuan (SC), northern and western Shandong (SD), and the junction of Hubei (HB) and Hunan (HN)); and (3) low-suitable habitats (slightly larger than the medium-suitable habitats, covering 7.1 × 10^5^ km^2^ (7.4%; Fig. 5b)), and it is distributed in Yunnan (YN), central Guangxi (GX), eastern and northern Guizhou (GZ), southern Shaanxi (SN), western and northern Henan (HA), and southern Hebei (HE)).

### RF model prediction of *M. azedarach* projected suitable habitats future changes

**Figure 6:**
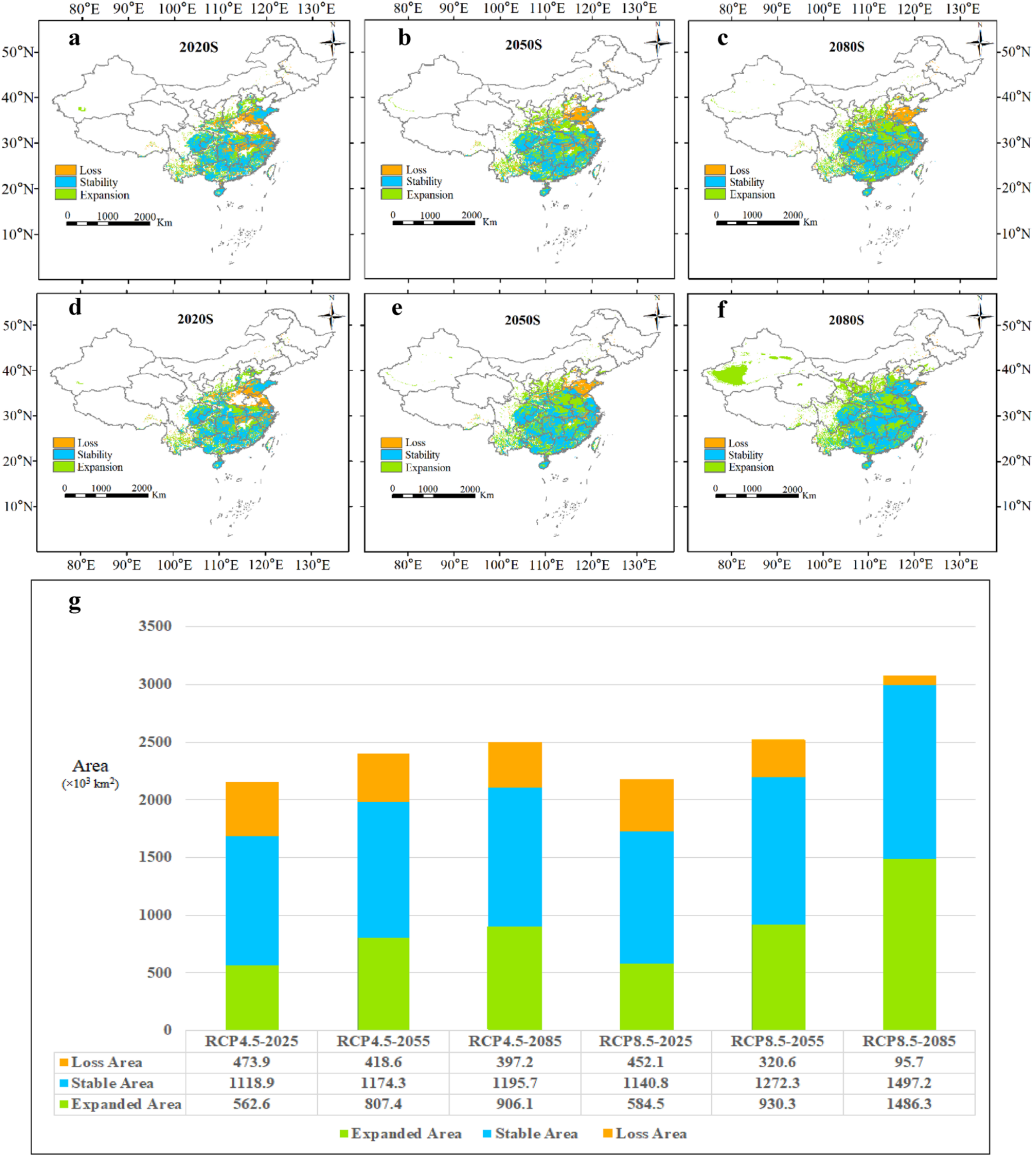
RF projected range changes for *M. azedarach* under RCP 8.5 and RCP 4.5 climate change scenarios (**a**–**f**) (**g** shows areas of habitat change). The map created in ArcMap 10.2 of the Environmental System Resource Institute, Icn. (https://www.esri.com/zh-cn/arcgis/products/arcgis-desktop/resources).

Future projections using the RF model with two different climate scenarios (RCP 8.5 and RCP 4.5) indicated a clear graphical expansion of *M. azedarach* in the future periods with an increasing magnitude over time (Fig. 6). The projected range increase was greatest under RCP 8.5 as compared to RCP 4.5 (Fig. 6). More specifically, the expanded area would increase by 562.6 × 10^3^ km^2^ and 584.5 × 10^3^ km^2^ by 2020s, 807.4 × 10^3^ km^2^ and 930.3 × 10^3^ km^2^ by 2050s, and 906.1 × 10^3^ km^2^ and 1486.3 × 10^3^ km^2^ by 2080s under the RCP4.5 and RCP8.5 scenarios, respectively (Fig. 6g). The main expanded area will be located in Yunnan (YN), Anhui (AH), Henan (HA), Shanxi (SX), Shaanxi (SN), central Guangxi (GX), central Jiangxi (JX), and northern Guizhou (GZ). Interestingly, based on the RCP8.5 climate scenario, Xinjiang (XJ) would see a larger magnitude of area expansion in 2080s (Fig. 6f). Additionally, the species stable range area showed the same change pattern as that of the expanded area (Fig. 6g). The main stable area included Guangdong (GD), Guangxi (GX), Guizhou (GZ), Hunan (HN), Chongqing (CQ), Fujian (FJ), Zhejiang (ZJ), Jiangsu (JS), southwestern Jiangxi (JX), and eastern Sichuan (SC) (Fig. 6a–f). Furthermore, the species area loss exhibited an opposite trend to that of expansion and stable range areas (Fig. 6f) and most of the loss area was mainly distributed in eastern coastal provinces near 30–38° N (e.g., Shandong (SD)) (Fig. 6).

## Discussion

### Model performance

Here, we used the AUC, Kappa, and OA to evaluate the performance of seven species range prediction models (GLM, GBM, MaxEnt, SVM, XGBoost, NBM, and RF) to predict *M. azedarach* contemporary and future ranges under two climate scenarios (RCP 8.5 and RCP 4.5). The results showed that RF and XGBoost were the top-performing models with RF being the best, while NBM and GLM were the low-performing with the NBM being the worst. Similarly, multiple lines of evidence support the superiority of the RF algorithm^26^. In a study in northern California, the GLM, ANN, RF and ME models were used to predict new occurrences for rare plants, and the results showed that RF provided the best prediction^27^. Akpoti et al. used BRT, GLM, MAXNT and RF algorithms to predict rice production suitability and the results showed that RF has better generalizability^28^. Silva et al. found the highest model quality for the RF and GAM algorithms when assessing the limitations of different species distribution models using the Azorean Forest as an example^29^. The RF is an ensemble machine-learning model that could handle data with multi-dimensional, non-linear relationships, high-order correlations, and missing values^30^. Additionally, the RF model is capable of avoiding the accuracy reduction problem caused by missing and noisy data in the training sample when predicting the relationship between a large number of predictor variables and the response variable^31^, attributes supporting the present study results. In contrast, while the NBM like RF is also a machine learning algorithm, it was proven to be not very sensitive to missing data, and the algorithm is relatively simple^32^. Studies have demonstrated that more complex species distributions models provided better predictive performance demonstrating the suitability of the RF model in processing complex high-dimensional data such as the data used in the present study^33^. Moreover, the NBM is a linear classifier and similar to the traditional linear statistical methods, all are insufficient in revealing the complex relationship among environmental variables^34^. In our case, the two linear models, GBM and GLM, demonstrated this with their poor predictive power. Additionally, we observed that the prediction accuracy of the XGBoost was very close to that of RF as the XGBoost has good generalization performance^35^. Although, previous studies have shown that MaxEnt, SVM, and GBM models performed well in simulating species suitability distribution^36,37^, our results have shown that the prediction accuracy of these models was intermediate relative to the performance of the seven tested models. These phenomena may indicate that species characteristics and sample size also have influence on the accuracy of species distribution models^38^.

### The importance of climate variables

Our study along with several others^39–41^ were based on the assumption that species distribution is mainly determined by climate^42,43^. It is well documented that climatic factors are key elements for most species’ population regeneration^44^. Here, our results indicated that temperature-associated climate factors have greater influence on *M. azedarach* suitable habitats than precipitation factors. Specifically, five of the top six contributing climatic variables were related to low temperature (MCMT, NFFD, and DD < 18) and continentality (TD), with MCMT contributing the most. This shows that low temperature was the main climatic factor that restricted *M. azedarach* suitable habitat, which is consistent with previous studies, as low-temperature stress imparted a negative impact on plant physiological and biochemical responses (e.g., plant membrane system disorder, photosynthetic rate decline, harmful active oxygen increased, and osmotic adjustment substances increase)^45^. The extension of the number of frost-free days (NFFD) was beneficial to increasing *M. azedarach* seed size and quality, thereby improving the survival rate^46^. In addition, MAP also influences the distribution of *M. azedarach* under certain TD conditions, as a warm and humid climate favors the growth and biomass accumulation of *M. azedarach*^47^. Xu et al. also confirmed that the ground diameter of *M. azedarach* tended to increase with increasing precipitation^48^.

### Range shift in response to climate change

Our study showed that *M. azedarach* would benefit from the anticipated climate change. More specifically, we found the RCP 8.5 scenario to be more favorable for the species habitat suitability expansion as compared to the RCP 4.5 scenario (Fig. 5g). The RCP 8.5 scenario predicted a greater increase in future temperature warming and precipitation, providing climatic conditions favorable to the species growth^46^. From the species geographic range change point of view, it is expected that the future suitable habitat distribution to expand north- and west-ward. Compared with the RCP4.5 scenario, the predicted trend of suitable habitats changes of the RCP8.5 scenario was more significant in the plateau area near 40° N (Fig. 5), including the Xinjiang Tarim Basin (RCP8.5) (Fig. 5f). Under the RCP4.5 and RCP8.5 scenarios, the future temperature is envisaged to rise by 1.4–1.8 and 2.0–3.7 °C, respectively, making high latitude areas warmer, resulting in a contemplated rise of mountains tree line, which would ultimately provide the species with a potential of geographic range expansion^49^. At the same time, we noted that the suitable habitat in the Shandong region would experience substantial range loss (Fig. 5), caused by a drastic change in climatic conditions from mainly dry continental airflow with little precipitation to a future warmer climate associated with intensified precipitation reduction^50^. Additionally, the impact of subtropical high pressure could not be overlooked as the Shandong is often affected by sinking air currents with long periods of high temperature and low precipitation. This subtropical high pressure is expected to gradually moved northward, followed by anticipated clear trend of northward movement associated with precipitation pattern change in the Shandong^51^. To a certain extent, the contemplated climate changes are expected to exacerbate the dryland climate in the Shandong, creating predominantly drought conditions that is unsuitable for the drought-intolerant *M. azedarach*^52^.

### Management strategies

Rapid climate change causes most tree populations to exist in unsuitable environmental conditions, threatening their growth and survival and even leading to population extinction^53^. Some tree species adapted to the new climatic conditions by migrating to the same environmental gradient or evolving^54^; however, other tree species would benefit from climate change^55^. *M. azedarach* belongs to those species who would benefit from future climate change leading to anticipated range expansion. The wide distribution of *M. azedarach* harbours abundant phenotypic variation and most of the species phenotypic diversity is mainly distributed in the southwest and south regions and to a lesser extent in other regions^56^. It is worth noting that if a widely distributed species could not track the changing climate due to long-term local adaptation, they would become more vulnerable^57^. Therefore, to prevent this uncertainty, we suggest taking proactive in-situ conservation measures for Yunnan, Guizhou, Sichuan, Guangdong, and Guangxi regions, as they are rich in phenotypic diversity which will help in coping with future environmental uncertainty^58^. Assisted migration initiatives should apply to presently unsuitable habitats that are expected to be suitable in the future. For example, the northern regions of Jiangxi, Hubei, Anhui, Henan, and areas near 40° N are reasonable targets for assisted migration conservation measures^59^. We recommend for areas that would be negatively affected by future climate as Shandong, taking ex-situ measures through establishing botanical gardens and seed banks in suitable habitats to protect their resources. Therefore, analyzing the ex-situ target areas’ climate ecology could provide reference for breeding programs and seed transfer guidelines/polices. At the same time, we suggest that other biological factors along with climate should also be considered in the species future research, such as species interaction (allelopathy, soil nutrient competition), land-use change (bio-energy farmland expansion), and the influence of human activities^60,61^, these factors collectively affect the contemporary and future distribution of *M. azedarach*.

## Conclusion

Here, we used three common model accuracy evaluation indicators to compare the suitability of seven data mining techniques for predicting *M. azedarach* distribution. The RF model, with its strong robustness and stability, provided the highest accuracy in establishing a climate niche model. Based on this model, maps of contemporary and future suitable habitats were developed. The RF prediction results indicated that *M. azedarach* would benefit from future climate change through range expansion and this has tendency towards north- and west-ward expansion. In order to maximize the species protection and development, we recommend taking a proactive in-situ conservation measures to conserve genetic variation for adaptation to uncertainties and ex-situ conservation to protect genetic resources under risk, and assisted migration to better use the areas with good potential in future climates.

## Supplementary Information

Supplementary Information.

## Data Availability

The datasets used and/or analyzed during the current study are available from the corresponding author on reasonable request.
