# Supplementary material for: Predicting suitable habitats of Melia azedarach L. in China using data mining
Source: Sci Rep. 2022 Jul 23;12:12617. doi: 10.1038/s41598-022-16571-y (PMC9308798; doi:10.1038/s41598-022-16571-y)
Supplement: Supplementary file 1 — Supplementary Information. [file 41598_2022_16571_MOESM1_ESM.docx]

**Table S1 List of the climate variables used in Melia azedarach climate change modeling (source: Wang et al., 2017)**

| **Code** | **Description** | **Code** | **Description** |
| --- | --- | --- | --- |
| MAT | mean annual temperature (℃) | DD>5 | degree-days above 5℃, growing degree-days |
| MWMT | mean warmest month temperature (℃) | DD<0 | degree-days below 0℃, chilling degree-days |
| MCMT | mean coldest month temperature (℃) | NFFD | the number of frost-free days |
| TD | temperature difference between MWMT and MCMT, or continentality (℃) | PAS | precipitation as snow (mm) between August in  previous year and July in current year |
| MAP | mean annual precipitation (mm) | EMT | extreme minimum temperature over 30 years |
| EXT | extreme maximum temperature over 30 years | Eref | Hargreaves reference evaporation |
| AHM | Annual heat:moisture index(MAT+10)/(MAP/1000)) | CMD | Hargreaves climatic moisture deficit |
| DD<18 | degree-days below 18°C | DD>18 | degree-days above 18°C |

**Table S2 List of variance inflation factors for 16 climate variables**

| **Variable** | **VIF** | **Variable** | **VIF** |
| --- | --- | --- | --- |
| MAT | 9830.55 | DD>5 | 3292.14 |
| MWMT | 2391.78 | DD<0 | 51.13 |
| MCMT | 12266.95 | NFFD | 111.07 |
| TD | 10865.61 | PAS | 2.54 |
| MAP | 7.18 | EMT | 273.64 |
| EXT | 19.24 | Eref | 38.34 |
| AHM | 2.92 | CMD | 8.49 |
| DD<18 | 5708.69 | DD>18 | 1813.33 |


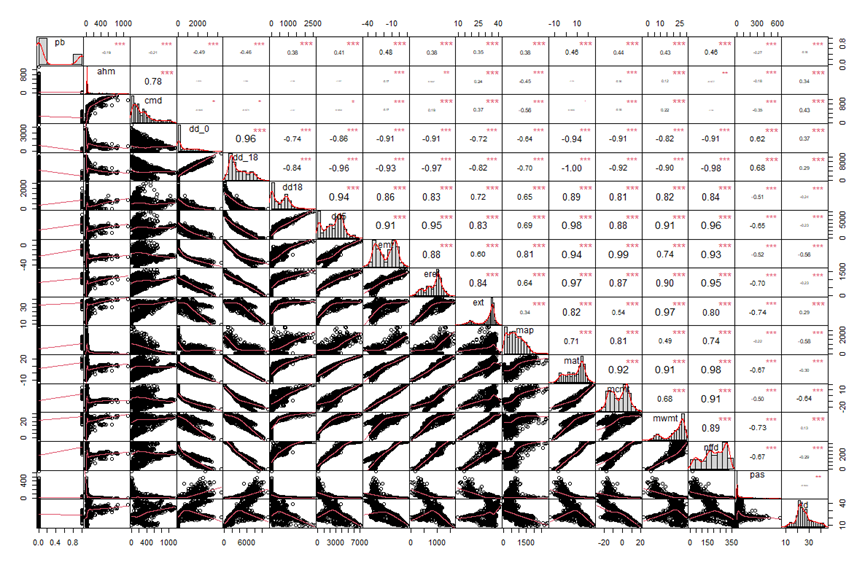


**Figure S1 Correlation matrix of the 16 predictors**
